# Supplementary material for: In Vitro Biodegradation of a-C:H:SiOx Films on Ti-6Al-4V Alloy
Source: Materials (Basel). 2022 Jun 15;15(12):4239. doi: 10.3390/ma15124239 (PMC9231245; doi:10.3390/ma15124239)
Supplement: Supplementary file 1 [file materials-15-04239-s001.zip › materials-1727788-supplementary.pdf]

## Supplementary Materials

*A*

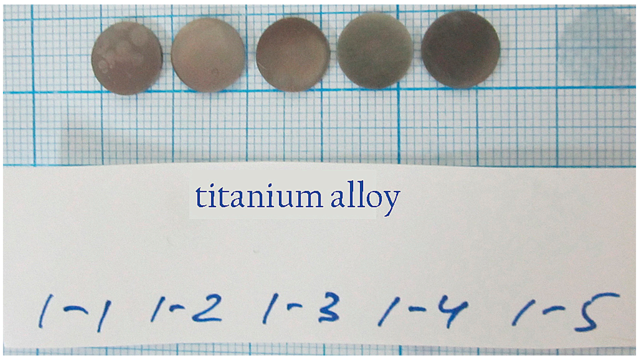


*B*

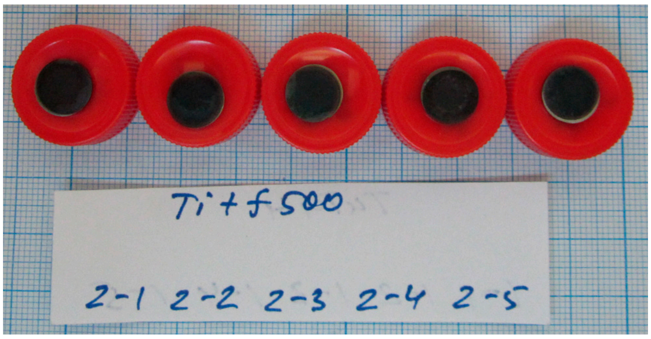


*C*

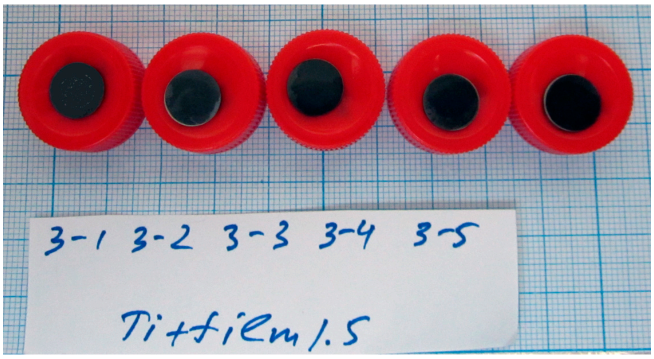


*D*

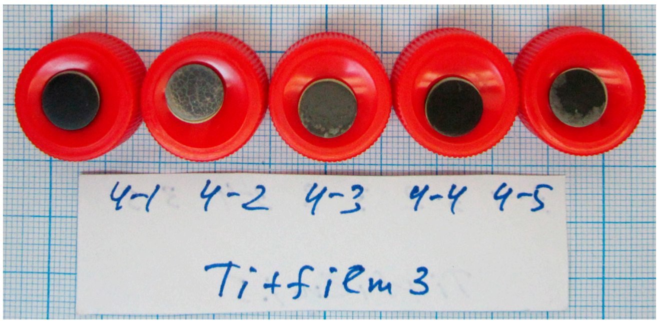


**Figure S1.** Ti-6Al-4V substrate surface after five-week *in vitro* immersion in 0.9% NaCl solution: *A* – uncoated, *B* – 0.5 µm, *C* – 1.5 µm, *D* – 3 µm film thickness.

**Table S1.** $Na^+$ concentrations (mmol) in 0.9% NaCl solution during five-week in vitro contact with test substrates, *Me(Q1; Q3)*, $n_1$=15.

| Group number | Substrates | $Na^+$ concentration in solution, mmol | | | | |
|---|---|---|---|---|---|---|
| | | After 1 week | After 2 weeks | After 3 weeks | After 4 weeks | After 5 weeks |
| 1 | 0.9 % NaCl reference solution | 155.1 (136.5; 158.2) | 155.8 (154.5; 156.4) | 155.1 (153.7; 156.0) | 156.5 (155.5; 157.3) | 156.1 (154.6; 157.1) |
| 2 | Uncoated Ti-6Al-4V | 157,7 (156.5; 157.9) | 157.4 (156.7; 158.2) | 156.2 (155.2; 156.6) | 157.1 (122.8; 157.5) | 158.6 (158.0; 159.5) |
| 3 | a-C:H:$SiO_x$-coated 0.5-µm thick film | 153.9 (94.9; 157.5) | 157.4 (157.3; 157.9) | 158.2* (157.7; 158.7) $P_{U1}$<0.05 | 158.2 (158.0; 158.4) $P_{U1}$<0.05 | 155.1*** (111.5; 158.6) |
| 4 | a-C:H:$SiO_x$-coated 1.5-µm thick film | 158.2 (157.3; 159.5) | 156.9 (156.9; 158.3) | 158.7 (158.4; 159.1) | 158.0 (157.7; 158.8) $P_{U1,2}$<0.05 | 157.7 (157.4; 158.0) |
| 5 | a-C:H:$SiO_x$-coated 3-µm thick film | 158.6 (158.5; 159.4) | 156.5 (154.8; 156.8) | 157.1 (156.9; 157.9) $P_{U1}$<0.05 | 157.9 (157.3; 158.4) | 158.9 (158.5; 158.9) |

**Table S2.** $Cl^-$ concentrations (mmol) in 0.9% NaCl solution during five-week in vitro contact with test substrates, *Me(Q1; Q3)*, $n_1$=15.

| Group number | Substrates | $Cl^-$ concentration in solution, mmol | | | | |
|---|---|---|---|---|---|---|
| | | After 1 week | After 2 weeks | After 3 weeks | After 4 weeks | After 5 weeks |
| 1 | 0.9 % NaCl reference solution | 129.1 (114.2; 133.3) | 129.8 (129.4; 130.1) | 129.3 (129.0; 130.8) | 130.7 (130.6; 131.0) | 131.1 (130.2; 131.5) |
| 2 | Uncoated Ti-6Al-4V | 132.6 (131.0; 132.6) | 131.2 (131.0; 132.2) | 131.1 (130.3; 131.2) | 131.3 (130.5; 131.5) | 132.2 (132.1; 132.3) |
| 3 | a-C:H:SiOx-coated 0.5-µm thick film | 131.1 (128.2; 131.5) | 131.4 (131.0; 131.6) | 131.7 (131.1; 132.1) $P_{U1}$<0.05 | 131.5 (131.1; 132.0) | 129.5 (89.4; 132.3) |
| 4 | a-C:H:SiOx-coated 1.5-µm thick film | 132.3 (132.1; 133.4) $P_{U1,3}$<0.05 | 130.9* (130.8; 131.7) | 132.4 (132.4; 133.2) | 132.1** (131.4; 133.2) | 132.5 (131.9; 132.5) |
| 5 | a-C:H:SiOx-coated 3-µm thick film | 132.8 (132.6; 133.1) $P_{U3}$<0.05 | 130.9 (130.7; 130.9) | 131.6** (131.0; 132.1) $P_{U1}$<0.05 | 131.5** (131.3; 132.0) | 132.3** (132.1; 132.5) |

*Note*: here and in Table S1 $n_1$ is the number of measurements in each group. Significant differences were determined: $P_{U1}$-$P_{U4}$ < 0.05 vs. the corresponding group number with the Mann-Whitney U test; $P_T$ < 0.05 vs. 1st week (*), 2nd week (**), and 3d week (***) with the Wilcoxon signed-rank test.
